# Investigating the Effect of Playing Different Defensive Styles and Court Sizes on Physical, Perceived, and Technical Demands in Basketball Small-Sided Games

**DOI:** 10.5114/jhk/202929

**Published:** 2025-04-30

**Authors:** Milos Nikolic, Zoran Milanovic, Pierpaolo Sansone, Henrikas Paulauskas, Paulius Kamarauskas, Daniele Conte

**Affiliations:** 1 Faculty of Sport and Physical Education, University of Niš, Niš, Serbia.; 2 Science and Research Centre Koper, Institute for Kinesiology Research, Koper, Slovenia.; 3 Incubator of Kinanthropology Research, Faculty of Sports Studies, Masaryk University, Brno, Czechia.; 4 Department of Education and Sport Sciences, Pegaso Telematic University, Naples, Italy.; 5 Institute of Sport Science and Innovations, Lithuanian Sports University, Kaunas, Lithuania.; 6 Education Academy, Vytautas Magnus University, Kaunas, Lithuania.; 7 Department of Movement, Human and Health Sciences, University of Rome “Foro Italico”, Rome, Italy.; 8 Department of Coaching Science, Lithuanian Sports University, Kaunas, Lithuania.

**Keywords:** external load, internal load, game-based conditioning drills, game-related statistics

## Abstract

This study aimed to determine the effects of different defensive styles (i.e., man-to-man vs. zone) and court sizes (full vs. half) on physical [PlayerLoad^TM^ (PL), total jumps and jumps in low (<20 cm), medium (21–40 cm), and high (>41 cm) bands, the number of and distance covered during accelerations and decelerations divided in high (>2 m∙s^−2^) and low intensity (<2 m∙s−2) bands], perceived [rating of perceived exertion (RPE)] and technical (total, scored, missed and % of made shots, rebounds, steals, assists, turnovers, and blocks) demands during basketball small-sided games (SSGs). Ten semi-professional male basketball players (age: 20.4 ± 2.1 years; stature: 189.4 ± 8.1 cm; body mass: 77.4 ± 8.4 kg) from the same basketball team participated in this study. Players were asked to play four 5 vs. 5 SSG typologies in randomized order: 1) half-court man-to-man defense, 2) half-court zone defense, 3) full-court man-to-man defense, and 4) full-court zone defense. No significant interaction (p > 0.05) between two independent variables was observed for physical demands. An effect of court size was found for most of the physical demand measures (except jumps) with higher values (p < 0.05) found in full court SSGs. The defensive style had an effect (p < 0.05, trivial-to-small) on total distance and low-intensity accelerations and decelerations. No effects were evident for the independent variables on the RPE and technical demands. Coaches should design full court SSGs when aiming at increasing players’ physical demands. Differently, similar physical, perceived and technical demands should be expected when playing man-to-man or zone defense during SSGs.

## Introduction

Small-sided games (SSGs) are a popular and effective training methodology adopted in basketball (Clemente, 2016; O’Grady et al., 2020). Basketball coaches use regularly SSGs to increase players’ physical capacity and technical skills while reproducing playing scenarios similar to the official games (Arslan et al., 2022; Clemente, 2016; Li et al., 2024; O’Grady et al., 2020). The manipulation of various contextual factors has been shown to modulate the physical and technical demands of SSGs. For instance, the number of players involved (Conte et al., 2016; de Souza et al., 2024a), rule changes (Conte et al., 2015; Ferioli et al., 2020), players’ rotation status (Sansone et al., 2023) and training regimes (Sansone et al., 2019) have been previously indicated as some of the main constraints which can be manipulated to determine the physical and technical demands of basketball SSGs.

In addition to these constraints, basketball coaches usually implement various defensive styles during basketball training and games. Changing the type of defensive styles can determine changes in the momentum of a basketball game and disrupt the offensive flow and efficiency of the opponent teams. For this reason, coaches dedicate time during training to prepare their teams for these different defensive scenarios. Consequently, it is essential for basketball research to analyse in detail the characteristics of different defensive styles. A recent investigation (Qarouach et al., 2024) assessing the adoption of various defensive strategies (i.e., switch, drop and trap) on the middle pick-and-roll offensive action documented higher physical (i.e., PlayerLoad and ACC) and perceived loads measured via the rating of perceived exertion (RPE) adopting a trap defense compared to switch and drop defenses during 3 vs. 3 SSGs played on a half court in female basketball players. Although these previous investigations provided very interesting practical implications for basketball coaches, they focused only on man-to-man defensive styles, while coaches usually require players to perform also a different defensive strategy such as zone defense. To the best of our knowledge, only one previous investigation (Castillo et al., 2021) has considered the differences in physical loads during 5 on 5 drills while playing man-to-man or zone defense in professional basketball players, revealing unclear differences between defensive strategies in various physical load measures such as total distance covered, distance covered in different speed zones, distance covered while accelerating and decelerating, maximum speed, steps, jumps and player load (Castillo et al., 2021). However, it should be considered that this previous study (Castillo et al., 2021) limited the investigation to physical load measures, while no information was available about the effect of playing different defensive styles on the perceived and technical demands of SSGs. This information seems essential for basketball coaches to understand which defensive style is more demanding allowing a comprehensive understating of the demands produced. Therefore, a further analysis assessing also perceived and technical demands is warranted.

In addition to the defensive style, court size is another relevant constraint that has significant effects on the physical and technical profile of SSGs. Previous studies have focused on the effect of playing SSGs on various court sizes. Montgomery et al. (2010) found that playing 5 vs. 5 full-court 5 on 5 scrimmage drills produced higher physical demands than playing the same drills half-court (Montgomery et al., 2010). In addition, a more recent study by Sansone et al. (2023) found that the court area per player influenced peak but not average physical demands imposed during game-based conditioning. Considering technical demands, a higher number of technical actions was evident when playing half-court compared to full-court in drills encompassing 2 vs. 2, 3 vs. 3 and 4 vs. 4 players (Atli et al., 2013; Klusemann et al., 2012). Nevertheless, to the best of our knowledge, no study has examined the combined effects of different defensive styles (man-to-man; zone) and court sizes (half-court; full-court) on the physical, perceived and technical demands in basketball SSGs. Understanding the impact of different defensive styles on half- and full-court SSGs would allow coaches to better design and implement training drills that replicate competition scenarios, ultimately improving the team’s readiness for games. Therefore, the aim of this study was to determine the effects of different defensive styles and court sizes on physical, perceived and technical demands during basketball SSGs.

## Methods

### Participants

Ten semi-professional male basketball players (mean ± SD; age: 20.4 ± 2.1 years; stature: 189.4 ± 8.1 cm; body mass: 77.4 ± 8.4 kg), from the same basketball team competing in the Lithuanian 3^rd^ division [Regionų Krepšinio Lyga (RKL)], participated in this study. Players were randomly divided into two teams based on playing positions and skill levels according to coaching staff evaluations to ensure a between-team balance (Sansone et al., 2019). Selection criteria included players who did not suffer from any injury during the last three months before the commencement of the study, according to self-reported data obtained through structured interviews. All players participated in four to five training sessions per week, at the same time of the day (i.e., 18.00–19.30) in the same sports hall and played one competitive game per week. All participants were over 18 years old and voluntarily participated in this study. They were informed about the purpose, risks, and benefits of this study and gave their informed consent in accordance with the Declaration of Helsinki. The study was approved by the ethics committee of the Lithuanian Sports University, Kaunas, Lithuania (protocol code: BEK-KTV(M)-2019-161; approval date: 11 March 2019).

### Design

Data were collected across two weeks of the in-season period, during which players were involved in four training sessions (i.e., two sessions per week). Before the commencement of the study, players were familiarized with SSG procedures and the adopted questionnaires. The four SSG typologies were played at the beginning of the training sessions (on Tuesdays and Thursdays) following a standardized warm-up, and randomized in the following order: 1) half-court man-to-man defense, 2) half-court zone defense, 3) full-court man-to-man defense, and 4) full-court zone defense. Regarding man-to-man defense, players were required to defend on one opponent during each SSG. Differently, during SSGs encompassing a zone defense, players were required to play a 2–3 zone defense, which represented one of the most adopted defensive strategies in basketball. All SSGs were preceded by a standardized 15–20-min warm-up consisting of low-intensity running, striding, and dynamic stretching. All training sessions were designed, directed, and supervised by the team’s coach on a regular-sized basketball court. Each SSG encompassed two 5-min bouts interspersed by a 1-min active rest interval in which participants were walking and were able to drink ad libitum. In each SSG, the International Basketball Federation rules were adopted, including the 24-s shot-clock, while time-outs and free throws were excluded. Moreover, after fouls, the game started with a throw-in from out-of-bounce from the side or baseline. When a shot was scored, the opponent team was re-positioning outside the 3-point line and was required to re-start playing as fast as possible.

### Procedures

#### Physical Demands

Physical demands were assessed using a local positioning system (LPS) (Catapult, Clear Sky T6, Catapult Innovations, Melbourne, Australia), which has been shown to have acceptable validity in measuring movements in court-based sports (Conte et al., 2022; Serpiello et al., 2018). Specifically, players were fitted with devices including an accelerometer, a gyroscope, and a magnetometer, and recording at 100 Hz. The following data deriving from inertial movement units (IMU) were collected: PlayerLoad^TM^ (PL), which was a measure calculated as the accumulated rate of change in acceleration across the three vectors (X, Y, Z) applying an established formula, and its relative value per minute (PL•min^−1^). Moreover, total distance covered and the number of total jumps and jumps in low (<20 cm), medium (21–40 cm), and high (>41 cm) bands were registered. Additionally, the number of and distance covered during accelerations and decelerations divided in high-intensity (>2 m∙s^−2^) and low-intensity (<2 m∙s^−2^) bands were assessed.

#### Perceived Demands

The internal load for each player was determined by RPE scores, which have been widely used to assess training loads in basketball players (Lupo et al., 2017; Sansone et al., 2023). Data were collected few minutes after each SSG using the CR-10 scale. Accordingly, every player answered the following question: “How was your training session?”, and value 0 was “rest”, while value 10 was “maximal”.

#### Technical Demands

Each SSG was recorded by two fixed cameras (Panasonic 4k Ultra Camcorder HC-WXF995M, Kadoma, Japan) positioned on the stands 3 m behind the basketball court baseline. After data collection, all videos were downloaded on a personal computer and analyzed through Kinovea software (v0.8.27; Kinovea Open Source Project, www.Kinovea.org), which has been used and described in previous basketball studies (Conte et al., 2015, 2016, 2017). Technical demands were assessed through a notational analysis technique by one researcher with ten years of basketball experience as a video analyst, and showed acceptable intra-tester reliability (intra-class correlation coefficient = 0.98, 95% CI: 0.98 to 0.99; coefficient of variation: 7.4%). The technical demand variables considered were the following: total shots, scored shots, missed shots, the percentage of made shots, rebounds, steals, assists, turnovers, and blocks. These were chosen as they represent key technical performance indicators in basketball (Sampaio et al., 2006; Zhou et al., 2024).

### Statistical Analysis

For physical demands, descriptive statistics were calculated as mean and standard deviation (± SD), while median and interquartile range (± IQR) were also calculated for perceived demands and technical demands. Normality and homogeneity of variance were assessed using the Kolmogorov-Smirnov test and the Shapiro-Wilk test with results showing physical demand data were normally distributed. Therefore, a two-way (defensive style x court size) ANOVA with repeated measures was used. Successively, effect sizes (Cohen’s *d*) were calculated to determine the magnitude of the difference between the defensive style and the court size, with values interpreted as: 0–0.19 = trivial, 0.20–0.59 = small, 0.60–1.19 = moderate, 1.20–1.99 = large, >2.0 = very large (Hopkins et al., 2009). Regarding the analyses of the RPE and technical demands, a Friedman test was used to compare the four conditions (i.e., man-to-man defense in a half court, zone defense in a half court, man-to-man defense in a full court and zone defense in a full court). For any statistically significant difference, a Wilcoxon signed-rank test was used with Bonferroni correction. For some technical actions (i.e., rebounds, assists, steals, turnovers, and blocks) only descriptive statistics were reported due to a low frequency of occurrence. All statistical analyses were performed using SPSS software (SPPS Inc. 23 Chicago, USA) and statistical significance was set at *p* ≤ 0.05.

## Results

Differences in the defensive style and court size for each external load measure are shown in Table 1. No significant interactions (*p* > 0.05) between the defensive playing style and court size were observed for any physical demand measure. An effect (*p* < 0.05) of court size was found for Total PL, PL•min^−1^, maximum velocity, total distance, distances for highDEC and lowDEC, lowACC, highACC, and the number of highDEC, lowDEC, lowACC, and highACC (Table 1), which were all higher in full-court than half-court (ES: moderate-very large). Moreover, an effect of the defensive style (*p* < 0.05) was found for total distance, and distances for lowDEC and lowACC, which tended to be higher in man-to-man than zone defense (ES: small–trivial).

**Table 1:** Differences in physical demands according to defensive styles and court sizes.

Physical load measures	Man-to-man		Zone			Court size			Defensive style		
	Full-court	Half-court		Full-court	Half-court		Full-court vs. Half-court			man-to-man vs. zone		
*UWB measures*	*mean ± SD*	*mean ± SD*		*mean ± SD*	*mean ± SD*		*p-value*	*ES (interpretation)*		*p-value*	*ES (interpretation)*	
Max velocity (m•s^−1^)	6.4 ± 0.5	4.8 ± 0.3		6.4 ± 0.4	4.7 ± 0.4		**<0.001**	3.31 (very large)		0.441	0.05 (trivial)	
Total distance (m)	1257.7 ± 42.9	884.0 ± 62.2		1207.4 ± 39.7	782.0 ± 65.5		**<0.001**	4.75 (very large)		**<0.001**	0.18 (trivial)	
HighDEC distance (m)	30.2 ± 4.1	24.9 ± 5.5		29.4 ± 5.4	23.1 ± 5.3		**0.001**	0.76 (moderate)		0.440	0.13 (small)	
LowDEC distance (m)	474.9 ± 30.0	315.6 ± 20.8		446.3 ± 29.9	273.4 ± 31.1		**<0.001**	4.82 (very large)		**<0.001**	0.20 (small)	
HighACC distance (m)	103.4 ± 19.3	77.9 ± 14.5		103.9 ± 14.3	68.2 ± 10.1		**<0.001**	4.06 (very large)		0.329	0.12 (trivial)	
LowACC distance (m)	648.9 ± 32.0	465.1 ± 36.4		626.0 ± 28.3	416.7 ± 32.3		**<0.001**	4.06 (very large)		**0.001**	0.17 (trivial)	
*Accelerometer-based measures*												
Total PL (AU)	155.2 ± 14.3	122.1 ± 9.6		152.5 ± 13.2	113.7 ± 12.2		**<0.001**	2.96 (very large)		0.167	0.14 (trivial)	
PL•min^−1^ (AU)	14.1 ± 1.3	11.8 ± 0.9		14.4 ± 1.2	11.0 ± 1.2		**<0.001**	2.28 (very large)		0.410	0.09 (trivial)	
HighDEC (n)	9.1 ± 3.2	6.5 ± 3.9		8.8 ± 2.2	5.0 ± 3.1		**0.003**	0.77 (large)		0.372	0.16 (trivial)	
LowDEC (n)	153.4 ± 10.8	168.6 ± 9.0		149.2 ± 10.9	170.5 ± 7.8		**<0.001**	−1.80 (large)		0.710	0.05 (trivial)	
HighACC (n)	10.4 ± 2.6	7.3 ± 2.0		11.3 ± 2.5	5.4 ± 2.6		**<0.001**	1.34 (large)		0.520	0.09 (trivial)	
LowACC (n)	155.0 ± 10.4	168.4 ± 8.6		153.4 ± 8.2	172.1 ± 7.9		**<0.001**	−1.15 (moderate)		0.709	−0.05 (trivial)	
Low Jumps (n)	5.7 ± 2.7	7.1 ± 4.5		7.2 ± 4.1	10.1 ± 3.6		0.082	−0.57 (small)		0.070	−0.48 (small)	
Medium Jumps (n)	6.2 ± 2.7	4.9 ± 2.1		7.2 ± 3.2	6.7 ± 3.4		0.333	0.31 (small)		0.135	−0.39 (small)	
High Jumps (n)	2.8 ± 2.2	2.2 ± 1.6		1.7 ± 1.6	2.1 ± 1.2		0.49	0.04 (trivial)		0.529	0.29 (small)	
Total Jumps (n)	14.7 ± 5.3	14.2 ± 5.9		16.1 ± 5.8	18.9 ± 6.8		0.815	−0.19 (trivial)		0.263	−0.49 (small)	

Note: bold font indicates significant differences at p < 0.05; UWB = ultrawide band; HighDEC = <−2 m•s−2; LowDEC = −0 to −2 m•s−2; HighACC = >2 m•s−2; LowACC = 0 to 2 m•s−2; PL = PlayerLoad; PL•min−1 = PlayerLoad per minute; Low Jumps = <20 cm; Medium Jumps = 20 cm to 40 cm; High Jumps = >40 cm

Considering the RPE, no significant differences (*p* = 0.139) were evident across the four conditions (median ± IQR; man-to-man half-court = 3 ± 0 AU; man-to-man full-court = 5 ± 2 AU; zone half-court = 3 ± 2 AU; zone full-court = 3 ± 2 AU; mean ± SD: man-to-man half-court = 3 ± 1 AU; man-to-man full-court = 4 ± 1 AU; zone half-court = 4 ± 1 AU; zone full-court = 4 ± 1 AU).

No differences for total shots (*p* = 0.579), scored shots (*p* = 0.638), nor missed shots (*p* = 0.332) were found (Table 2). The descriptive statistics for rebounds, assists, blocks, steals and turnovers are reported in Table 2.

**Table 2:** Median ± interquartile range, and mean ± standard deviation for technical demands according to defensive styles and court sizes.

	Man-to-man		Zone		*p*-value
Variable	Full-court	Half-court	Full-court	Half-court	
Total Shots (n)	3 ± 2; 3 ± 2	3 ± 4; 3 ± 2	4 ± 4; 4 ± 2	3 ± 4; 3 ± 2	0.579
Score Shots (n)	3 ± 1; 2 ± 1	2 ± 3; 2 ± 1	1 ± 1; 2 ± 1	2 ± 3; 2 ±2	0.638
Missed Shots (n)	1 ± 2; 1 ± 1	2 ± 2; 1 ± 1	2 ± 3; 2 ± 2	1 ± 1; 1 ± 1	0.332
Rebounds (n)	0 ± 1; 1 ± 1	1 ± 2; 1 ± 1	1 ± 5; 2 ± 2	0 ± 2; 1 ± 2	N/A
Assists (n)	1 ± 1; 1 ± 1	1 ± 2; 1 ± 1	1 ± 2; 1 ± 1	1 ± 2; 1 ± 1	N/A
Steals (n)	0 ± 1; 0 ± 1	0 ± 1; 0 ± 1	1 ± 1; 1 ± 1	1 ± 1; 1 ± 1	N/A
Turnovers (n)	1 ± 1; 1 ± 1	0 ± 1; 1 ± 1	2 ± 2; 1 ± 1	N/A; N/A	N/A
Blocks (n)	0 ± 0; 0 ± 0	0 ± 0; 0 ± 0	0 ± 0; 0 ± 0	0 ± 0; 0 ± 0	N/A

Note: N/A = not applicable due to the low number of observations

## Discussion

This study aimed to compare the physical, perceived and technical demands of SSGs played using two defensive styles (man-to-man vs. zone defense) and court sizes (half vs. full court). The main results revealed that most of the physical load measures were influenced by court size, while only total distance and distance covered during lowACC and lowDEC were influenced by the defensive styles adopted, however, with only trivial-to-small effect sizes. Furthermore, no effect of either the defensive style or court size was evident for the perceived demands and technical actions. Overall, these results suggest that coaches can modify mostly the court size to produce a different physical load during basketball SSGs, while man-to-man and zone defensive strategies posit substantially the same physical demands.

### Physical Demands

Monitoring the physical demand of basketball SSGs is fundamental to have clear information about the physical requirements during game-based training drills, one of the prominent training modes in basketball. This information can be used to optimize individual and team training programs across the basketball season. While most of previous studies synthetized in a systematic review (O’Grady et al., 2020) analyzing the effects of various constraints on SSGs adopted a man-to-man defense, to the best of our knowledge, this is the first study assessing the interaction of defensive styles and court sizes on physical demands during 5 vs. 5 SSGs.

Our results documented no interaction between the defensive style and court size for any of the investigated physical variable, suggesting that these two variables should not be considered jointly when aiming at modifying the physical loads imposed, but rather separately. Regarding the effects of defensive strategies, no differences were evident in most of the load measures monitored, and in those reporting significant difference (total distance and distance covered during lowACC end lowDEC) only trivial-to-small effect size was found. These results are in line with those reported in a previous investigation (Castillo et al., 2021) documenting unclear differences between man-to-man and zone defensive styles during game-based drills played 5 vs. 5 on a half court in professional basketball players. Moreover, our findings revealing the lack of differences in physical demands across various defensive styles adopted during SSGs are also in agreement with studies evaluating official basketball matches (Abdelkrim et al., 2010; Sampaio et al., 2016). Indeed, no differences in high-intensity actions (Abdelkrim et al., 2010) and total distance covered (Sampaio et al., 2016) were previously found in youth and semi-professional basketball players, respectively. Overall, our results confirm that the use of man-to-man and 2-3 zone defensive styles does not differ in physical demands during basketball game-based conditioning drills. These findings suggest that changing defensive strategy will not lead to meaningful variations in physical demands imposed on players, and that other constraints should be manipulated during training when aiming at modifying the external loads.

Differently than the defensive style, court size showed influence on all the assessed external load variables except for jumps, with overall higher physical demands in full-court compared to half-court SSGs (ES: moderate-to-very large). These outcomes overlap with previous investigations assessing the effect of court size on physical demands during basketball SSGs (O’Grady et al., 2020). Indeed, Bredt et al. (2020) showed that playing SSGs characterized by 3 vs. 3 on a full court resulted in more time spent in higher acceleration zones compared to 3 vs. 3 SSGs played on a half court in youth basketball players (ES: small-to-moderate). Similarly, Vazquez-Guerrero et al. (2020) found that 5 vs. 5 scrimmages played on a full court produced higher distance covered, peak speed, PL, high-intensity actions, high-intensity accelerations and decelerations compared to the same format played on a half court (ES: large-to-very large). Additionally, in a recent study (Sansone et al., 2023) assessing the effect of the court area per player on the peak external load per minute measured with 1-min rolling averages, it was found that a greater court area per player elicited higher peak physical demands compared to a smaller court area per player. Collectively, these findings suggest that court size is a key variable to manipulate in order to modify the physical demands encountered by basketball players during SSGs. Specifically, coaches can prefer half-court drills in order to decrease the overall external loads, and vice versa, select full-court drills in order to increase the external load.

### Perceived Demands

Monitoring perceived demands can provide fundamental information for basketball coaches and practitioners about the internal responses (i.e., internal loads) of players to the prescribed workload (i.e., external loads) (Jeffries et al., 2022). Our results showed no effect of playing man-to-man or zone defense during half- and full-court drills on RPE values during basketball SSGs. It is difficult to make comparisons with previous investigations since, to the best of our knowledge, no previous study had investigated these aspects combined. Previous research assessing the effect of court sizes on RPE values only documented contrasting results in youth basketball (Klusemann et al., 2012; Marcelino et al., 2016). While Klusemann et al. (2012) showed a moderate difference between SSGs played on a full (28 x 15 m) and a half court (14 x 15 m), Marcelino et al. (2016) found no difference (*p* > 0.05) in the RPE during SSGs played with the same court area per player, although with different settings (i.e., full court: 28 x 15 m vs. half court: 28 x 9 m). Our results also showed no differences between the SSGs played on a full court and a half court (14 x 15 m) either played with man-to-man or zone defense. A possible reason for this discrepancy could be the different statistical approach adopted across studies. Indeed, while in our investigation and in Marcelino et al.’s (2016) study a traditional frequentist statistical analysis was used with results categorized as significant or not based on the set alpha level of the *p*-value, Klusemann et al. (2012) adopted a magnitude-based inference approach, which was based on the differences between groups relative to a set smallest worthwhile change, which could lead to possible different statistical outcomes. Beside the inferential statistics adopted across studies, it should be noted that different results were also evident when considering descriptive analyses. Indeed, similar RPE values (i.e., ~7 AU for full-court and ~6 AU for half-court SSGs ranging between 10 and 16 min of live time) were found in previous studies (Klusemann et al., 2012; Marcelino et al., 2016), while our results showed lower RPE values, ranging between 3 and 4 AU (with 10 min of live time). A possible reason for the perceived lower RPE could be the different level of basketball players recruited for our study. Indeed, in our study, semi-professional players completed the SSGs, while previous investigations (Klusemann et al., 2012; Marcelino et al., 2016) involved youth basketball players (U17 and U19). Possibly, our semiprofessional players might be more experienced in competing during SSGs, and might have paced themselves during the drills, not perceiving the load as so exacerbating. Additionally, differences in players’ age (Groslambert and Mahon, 2006) and cultural differences (Rejeski, 1981) across studies involving different samples might lead to different interpretations and reporting of subjective scores, including those of perceived exertion. Overall, our results suggest that a combination of court size and the defensive style does not have a significant impact on the players’ RPE, suggesting other strategies to be used by basketball coaches to increase or decrease players’ perceived responses.

### Technical Demands

SSGs are a useful training method for the development not only of the physical and physiological characteristics of basketball players, but also for the development of their technical skills (de Souza et al., 2024b; Sansone et al., 2019). Nevertheless, players’ technical involvement in basketball SSGs has not been extensively studied for the constraints examined in our study. Specifically, no previous investigation had assessed the effect of playing different defensive strategies during SSGs on players’ technical demands, while only two investigations assessed the effects of different court sizes (Atli et al., 2013; Klusemann et al., 2012). Therefore, our results provide a unique insight, demonstrating no differences in technical demands across the four experimental sessions carried out (i.e., man-to-man half-court, zone half-court, man-to-man full-court, and zone full-court). However, it should be noted that only shooting actions (total, scored and missed shots) were considered for the inferential statistical analyses due to a low number of occurrence of the other technical actions (rebounds, assist, blocks, steals and turnovers) assessed. Intuitively, we would expect a higher number of shots corresponding to a reduced court size as found in previous studies (Atli et al., 2013; Klusemann et al., 2012). However, it should be considered that our SSGs were characterized by 5 vs. 5 drills, while previous investigations studied drills on a half and a full court with 2 vs. 2, 3 vs. 3 and 4 vs. 4 players. Playing SSGs with a reduced number of players allowed a considerably higher number of shots (average ranging between 12 and 18 shots per player for half-court drills and between 10 and 11 shots per player for full-court drills) (Atli et al., 2013; Klusemann et al., 2012) compared to our 5 vs. 5 drills (3 to 4 shots per player). The main reason possibly explaining the lower number of shots found in our study is the shot-clock duration, which was the full 24 s according to the official international basketball rules. Differently, previous game-based conditioning studies (Klusemann et al., 2012; Sansone et al., 2020) implemented changes in the shot-clock duration that directly affected the performance profile of SSGs, including a higher number of shots taken and ball possessions played with shorter clock duration (Klusemann et al., 2012; Sansone et al., 2020). Furthermore, the limited number of shots in our study diminishes the possibility of finding statistical differences across groups. Altogether, it could be suggested that playing 5 vs. 5 drills which narrowly replicate the real game scenarios (compared to drills with less players involved) does not allow a high number of technical actions, and the manipulation of other constraints such as a shot clock should be considered to increase the individual technical efforts of basketball players.

### Limitations and Future Directions

Although this study provides interesting insight for basketball coaches about the use of different defensive styles and court sizes during basketball SSGs, some limitations should be considered. First, no tactical demands were considered in our study, limiting the applicability of our results only to physical, perceived and technical demands. Additionally, only one zone formation (i.e., 2-3) was adopted, while different formations (1-3-1, 2-1-2, box and one, etc.) might lead to different outcomes. Moreover, our sample size was representative of one team composed of ten players, while a larger sample size might increase the results *generalizability*. Therefore, future studies should focus on the effects of playing various defensive strategies on different court sizes on tactical demands, explore whether different zone defenses could lead to varied physical, perceived and technical demands and involve a larger sample size.

## Practical Implications

From a practical standpoint, these results suggest basketball coaches adopting court size as the main variable to modify the physical load of their SSGs. In particular, it is recommended to use full-court drills to increase players’ physical loads, and half-court drills to reduce them. This could potentially help basketball coaches and practitioners in programming properly their training sessions and microcycles during various phases of the season. It is also suggested that basketball coaches would manipulate different constraints than those analyzed in this study when aiming at modifying the perceived exertion and technical demands in SSGs. Finally, from a physical, perceived and technical standpoint, playing man-to-man and zone defense with these SSG settings (i.e., 5 on 5 and with 10 min of live time duration) did not lead to meaningful differences, suggesting that coaches can use interchangeably these defensive strategies when aiming at developing players’ physical conditioning and technical skills using game-based scenarios.

## Conclusions

Playing man-to-man and zone defense did not impact players’ physical, perceived and technical demands. Differently, court sizes had an influence on physical demands, with full-court sizes inducing higher values for PL, total distance, maximum velocity, ACC and DEC. Coaches should adopt these results to design their SSGs, implementing full-court drills to increase their players’ physical loads or vice versa, preferring half-court drills when the goal is to reduce external loads. Moreover, similar physical, perceived and technical demands can be expected when playing SSGs encompassing either man-to-man or zone defense.
